# A Single Nucleotide Polymorphism Within Molybdenum Cofactor Sulfurase Gene Is Associated With Neuropsychiatric Conditions

**DOI:** 10.3389/fmolb.2020.540375

**Published:** 2020-09-24

**Authors:** Amin Safa, Mir Davood Omrani, Fwad Nicknafs, Alireza Komaki, Mohammad Taheri, Soudeh Ghafouri-Fard

**Affiliations:** ^1^Institute of Research and Development, Duy Tan University, Da Nang, Vietnam; ^2^Department of Immunology, Ophthalmology and ENT, School of Medicine, Complutense University, Madrid, Spain; ^3^Department of Medical Genetics, Shahid Beheshti University of Medical Sciences, Tehran, Iran; ^4^Neurophysiology Research Center, Hamadan University of Medical Sciences, Hamadan, Iran; ^5^Urogenital Stem Cell Research Center, Shahid Beheshti University of Medical Sciences, Tehran, Iran

**Keywords:** molybdenum cofactor sulfurase, rs594445, methamphetamine addiction, bipolar disorder, major depressive disorder

## Abstract

**Background:**

Molybdenum cofactor sulfurase (MOCOS) is an enzyme participating in purine metabolism. The aim of current study was to evaluate the role of a single nucleotide polymorphism (SNP) in the coding gene (rs594445) in mood disorders and methamphetamine addiction.

**Methods:**

This SNP was genotyped in 200 persons with methamphetamine addiction, 85 patients with bipolar disorder 1 (BP1), 78 patients with BP2, 33 patients with major depressive disorder (MDD) and 200 age-/sex-matched normal subjects using the tetra-primer amplification-refractory mutation system (ARMS)-PCR technique.

**Results:**

The rs594445 was associated with methamphetamine addiction in co-dominant model [A/A vs C/C: OR (95% CI) = 0.466 (0.252–0.864), *P*-value = 0.014; C/A vs C/C: OR (95% CI) = 0.641 (0.418–0.981), *P*-value = 0.04]. This SNP was also associated with this trait in dominant model [OR (95% CI) = 0.591 (0.398–0.879), *P*-value = 0.009]. The A allele of rs594445 had a protective role against methamphetamine addiction [A vs C: OR (95% CI) = 0.645 (0.48–0.866), *P*-value = 0.004]. The rs594445 was associated with BP1 in co-dominant model [C/A vs C/C: OR (95% CI) = 0.423 (0.230–0.778), *P*-value = 0.005]. However, the associations were insignificant in other inheritance models.

**Conclusion:**

Finally, there were no significant associations between the mentioned SNP and risk of BP2 or MDD in any inheritance model. The present project highlights the role rs594445 in two psychiatric conditions and implies the presence of common genetic factors for these disorders.

## Introduction

Molybdenum cofactor sulfurase (MOCOS) being encoded by the MOCOS gene transfers a sulfur atom to the molybdenum cofactor of xanthine dehydrogenase (XDH) and aldehyde oxidase (AOX1) and activates these enzymes (Ichida et al., 2001; Taheri et al., 2020). Mutations in human MOCOS gene have been associated with classical xanthinuria type II (Ichida et al., 2001). More recently, MOCOS has been shown to be down-regulated in adult nasal olfactory stem cells of subjects with autism spectrum disorder (ASD) compared with controls. Moreover, functional studies have shown that dysregulation in expression of this gene leads to synaptic flaws as well as hypersensitivity to oxidative stress (Feron et al., 2016). MOCOS has been shown to be expressed in several regions of the developing and adult human brain. Moreover, studies in mouse brain tissues have verified its expression in the hippocampus, cortex, cerebellum and brainstem (Feron et al., 2016).

This gene has a number of single nucleotide polymorphisms (SNPs) among them is rs594445. A previous study has provided clues for functionality of this SNP. This study has shown that renal transplant recipients having minor allele of this SNP needed lower doses of Azathioprine for proficient immunosuppression following allograft transplantation (Kurzawski et al., 2012). Moreover, this SNP slightly protects patients with inflammatory bowel disease against occurrence of adverse drug reactions to Azathioprine (Smith et al., 2009).

Based on the proposed role for MOCOS in neurodevelopment and neurotransmission (Feron et al., 2016), we aimed to identify its role in two psychiatric conditions namely mood disorders and methamphetamine addiction. So, we genotyped a probable functional SNP within this gene in these patients and healthy subjects to find any difference in allelic frequencies between groups.

## Materials and Methods

### Enrolled Individuals

A total of 200 persons with methamphetamine addiction, 85 patients with BP1, 78 patients with BP2 and 33 patients with MDD joined the current project. Moreover, 200 age-/sex-matched normal subjects were chosen as controls. Patients with mood disorders were referred to Hospitals affiliated with Shahid Beheshti and Hamadan Universities of Medical Sciences during 2016–2019. Addicted persons were referred to Niaz and Atinegar Addiction Treatment Centers, Mashhad, Iran during 2017–2019. Both groups were assessed and diagnosed using the 5th edition of Diagnostic and Statistical Manual of Mental Disorders (American Psychiatric Association, 2013). Individuals having any structural or metabolic brain disorders or medical conditions with psychiatric complications were excluded from the study. Persons with history of substance abuse were exempted from all groups excepting the “Addiction” group. Control subjects had no psychiatric conditions based on the results of a semi-structured interview. Written informed consent forms were signed by all enrolled persons. The study protocol was approved by the Ethics Committees of Shahid Beheshti Universities of Medical Sciences.

### Genotyping

Table 1 shows the general features of the selected SNP.

**TABLE 1:** General features of the rs594445 SNP.

SNP	Position	Minor allele	Minor allele frequency	Minor allele count	Type
rs594445	Chr 18:36251226	A	0.2228	1116	coding_sequence_variant, missense_variant

Blood samples were obtained from all aces and controls in EDTA-containing tubes. DNA was extracted from these samples using conventional salting out method. The alleles of rs594445 SNP were defined in each study participants using the tetra-primer amplification-refractory mutation system (ARMS)-PCR technique. Primers were designed using the Primer1 software (Collins and Ke, 2012). Samples were prepared using the Taq DNA Polymerase Master Mix RED (Amplicon, Denmark). Table 2 displays the information about the primers sizes, annealing temperature and the anticipated product sizes. Amplifications were executed using the following PCR program: denaturing phase at 95°C for 7 min; 30 cycles at 95°C for 30 s, 59°C for 40 s, 72°C for 1 min and an ultimate extension phase at 72°C for 7 min.

**TABLE 2:** Nucleotide sequence of primers and PCR conditions (F: forward, R: reverse, i: inner, o: outer).

Primer names	Primer sequences	Annealing temperature	Expected amplicon sizes	
Fo	ATTTTGTAACCAAACTTTTAGCAATTCAGA	59	Outer	358
Ro	GTGCAGTAGGTTTCTCCTAAAGGCT			
Fi (A)	GTCAACATTTTTTCGCCGTCCTTATA		Fi (A)	229
Ri (C)	TTGAGAGTTTGAACTTTGTTAGATCAAGTG		Ri (C)	184

### Statistical Assessments

All statistical tests were performed in R 3.2.2 software. Agreement/non-accordance with Hardy-Weinberg equilibrium (HWE) were evaluated in each study group using Chi-square test. Associations between the rs594445 allele/genotypes and mood disorders/methamphetamine addiction were evaluated in co-dominant, dominant, recessive and allelic models. Odds ratios (OR), 95% confidence intervals (95% CI) and *P*-values were computed to define the associations. *P*-values less than 0.05 were considered as significant.

## Results

### General Characteristics of Study Participants

General characteristics of study participants are listed in Table 3.

**TABLE 3:** General characteristics of study participants.

Variable	BPD1	BPD2	MDD	Control	Addiction	Control
Age (mean ± SD)	42 ± 4.2	42 ± 7.6	49 ± 1.0	47 ± 5.4	36.43 ± 9.79	37.76 ± 9.6
Gender						
Female	50	42	14	120	0	0
Male	35	36	19	80	200	200
Total	85	78	33	200	200	200

### Frequencies of Different Alleles of rs594445

Table 4 shows the allele frequencies of the different alleles of rs594445 in patients and controls.

**TABLE 4:** Frequencies of the different alleles of rs594445 in patients and controls.

	Addiction cohort		Mood disorders cohort			
Allele	Cases	Controls	BP1	BP2	MDD	Controls
C	285	246	108	107	47	246
A	115	154	62	49	19	154
Total	400	400	170	156	66	400

### Genotype Frequencies

Genotype frequencies of the rs594445 were in agreement with the HWE assumption in all groups of psychiatric patients and healthy subjects except for BP1 group (Table 5).

**TABLE 5:** Compliance of genotype frequencies of the rs594445 with HWE.

	Genotype	C/C	C/A	A/A	Total	*P*-value
Addiction cohort	Case	106	73	21	200	0.122932
	Control	80	86	34	200	0.193402
Mood disorders cohort	BP1	44	20	21	85	0.000006
	BP2	38	31	9	78	0.492974
	MDD	18	11	4	33	0.456803
	Control	80	86	34	200	0.193402

The rs594445 was associated with methamphetamine addiction in co-dominant model [A/A vs C/C: OR (95% CI) = 0.466 (0.252–0.864], *P*-value = 0.014; C/A vs C/C: OR (95% CI) = 0.641 (0.418–0.981), *P*-value = 0.04]. This SNP was also associated with this trait in dominant model [OR (95% CI) = 0.591 (0.398–0.879), *P*-value = 0.009]. The A allele of rs594445 had a protective role against methamphetamine addiction [A vs C: OR (95% CI) = 0.645 (0.48–0.866), *P*-value = 0.004]. Table 6 shows the detail analysis of associations between methamphetamine addiction and rs594445 SNP in four inheritance models.

**TABLE 6:** Associations between methamphetamine addiction and rs594445 SNP in four inheritance models.

Model	Genotype	Control		Case		OR (95% CI)	*P*-value
		A/A Number (%)	C/C Number (%)	A/A Number (%)	C/C Number (%)		
Co-dominant	A/A vs C/C	34 (0.170)	80 (0.400)	21 (0.105)	106 (0.530)	0.466 (0.252–0.864)	0.014
	C/A vs C/C	86 (0.430)	80 (0.400)	73 (0.365)	106 (0.530)	0.641 (0.418–0.981)	0.04
Dominant	C/A + A/A vs C/C	120 (0.600)	80 (0.400)	94 (0.470)	106 (0.530)	0.591 (0.398–0.879)	0.009
Recessive	A/A vs C/A + C/C	34 (0.170)	166 (0.830)	21 (0.105)	179 (0.895)	0.573 (0.32–1.027)	0.059
Allelic	A vs C	154 (0.385)	246 (0.615)	115 (0.288)	285 (0.713)	0.645 (0.48–0.866)	0.004

The rs594445 was associated with BP1 in co-dominant model [C/A vs C/C: OR (95% CI) = 0.423 (0.230–0.778), *P*-value = 0.005]. However, the associations were insignificant in other inheritance models (Table 7).

**TABLE 7:** Associations between BP1 and rs594445 SNP in four inheritance models.

Model	Genotype	Control		Case		OR (95% CI)	*P*-value
		A/A Number (%)	C/C Number (%)	A/A Number (%)	C/C Number (%)		
Co-dominant	A/A vs C/C	34 (0.170)	80 (0.400)	21 (0.247)	44 (0.518)	1.123 (0.582–2.166)	0.729
	C/A vs C/C	86 (0.430)	80 (0.400)	20 (0.235)	44 (0.518)	0.423 (0.230–0.778)	0.005
Dominant	C/A + A/A vs C/C	120 (0.600)	80 (0.400)	41 (0.482)	44 (0.518)	0.621 (0.373–1.035)	0.067
Recessive	A/A vs C/A + C/C	34 (0.170)	166 (0.830)	21 (0.247)	64 (0.753)	1.602 (0.866–2.965)	0.132
Allelic	A vs C	154 (0.385)	246 (0.615)	62 (0.365)	108 (0.635)	0.917 (0.632–1.33)	0.648

Finally, there were no significant associations between the mentioned SNP and risk of BP2 or MDD in any inheritance model (Tables 8, 9).

**TABLE 8:** Associations between BP2 and rs594445 SNP in four inheritance models.

Model	Genotype	Control		Case		OR (95% CI)	*P*-value
		A/A Number (%)	C/C Number (%)	A/A Number (%)	C/C Number (%)		
Co-dominant	A/A vs C/C	34 (0.170)	80 (0.400)	9 (0.115)	38 (0.487)	0.557 (0.243–1.278)	0.164
	C/A vs C/C	86 (0.430)	80 (0.400)	31 (0.397)	38 (0.487)	0.759 (0.432–1.333)	0.337
Dominant	C/A + A/A vs C/C	120 (0.600)	80 (0.400)	40 (0.513)	38 (0.487)	0.702 (0.415–1.188)	0.19
Recessive	A/A vs C/A + C/C	34 (0.170)	166 (0.830)	9 (0.115)	69 (0.885)	0.637 (0.29–1.398)	0.26
Allelic	A vs C	154 (0.385)	246 (0.615)	49 (0.314)	107 (0.686)	0.732 (0.494–1.084)	0.12

**TABLE 9:** Associations between MDD and rs594445 SNP in four inheritance models.

Model	Genotype	Control		Case		OR (95% CI)	*P*-value
		A/A Number (%)	C/C Number (%)	A/A Number (%)	C/C Number (%)		
Co-dominant	A/A vs C/C	34 (0.170)	80 (0.400)	4 (0.121)	18 (0.545)	0.523 (0.165–1.660)	0.265
	C/A vs C/C	86 (0.430)	80 (0.400)	11 (0.333)	18 (0.545)	0.568 (0.253–1.277)	0.168
Dominant	C/A + A/A vs C/C	120 (0.600)	80 (0.400)	15 (0.455)	18 (0.545)	0.556 (0.265–1.166)	0.12
Recessive	A/A vs C/A + C/C	34 (0.170)	166 (0.830)	4 (0.121)	29 (0.879)	0.673 (0.222–2.041)	0.48
Allelic	A vs C	154 (0.385)	246 (0.615)	19 (0.288)	47 (0.712)	0.646 (0.365–1.141)	0.13

## Discussion

In the present research project, we evaluated associations between rs594445 and mood disorders as well as methamphetamine addiction. This missense polymorphism may change enzyme activity of MOCOS and participate in the pathophysiology of human disorders. Although most of previous studies assessed its contribution in purine metabolism (Ichida et al., 2001), more recent studies reported expression of this gene in brain tissues and its participation in a neuropsychiatric condition namely ASD (Feron et al., 2016).

The rs594445 was associated with methamphetamine addiction in co-dominant, dominant and allelic models. Notably, the minor allele of rs594445 had a protective role against methamphetamine addiction. This allele might change MOCOS activity, and result in slower thiopurine metabolism (Kurzawski et al., 2012). However, the relevance of this altered enzymatic activity with methamphetamine addiction needs to be clarified. A previous study has shown changes in tryptophan and purine metabolism in cocaine-dependent individuals compared with controls (Patkar et al., 2009). Another study has indicated the effects of methamphetamine on the systemic metabolism in animal subjects (Zheng et al., 2014). The differences in metabolic pathways between carriers of different alleles of the rs594445 SNP might result in accumulation of specific metabolites following methamphetamine abuse leading to aversion. The similar mechanism has been explained for genetic factors that determine alcohol dependence. For instance SNPs within gene coding alcohol and aldehyde dehydrogenases may determine alcohol-dependence risk vs aversive response (Mayfield et al., 2008). However, such mechanisms have not been clarified for other substances which are subjects of addiction or abuse.

Besides, the rs594445 was associated with BP1 but not with BP2. The association between disturbed purine metabolism and mania has been acknowledged about one century ago (Kraepelin, 1921). Moreover, higher purinergic turnover has been suggested as a possible contributing factor in the pathogenesis of mania (Machado-Vieira et al., 2002). However, the reason for different pattern of association between BP1 and BP2 in our study is not clear. Future studies are needed to replicate these results in higher sample sizes of BP 1 and BP 2 patients and assess any possible difference.

Finally, there were no significant associations between the mentioned SNP and risk of BP2 or MDD in any inheritance model. This was an unexpected result as a previous study reported altered purine metabolism in MDD patients (Ali-Sisto et al., 2016). The mentioned study has shown decreased levels of inosine and guanosine, and elevated concentrations of xanthine in MDD patients compared with controls (Ali-Sisto et al., 2016). Future studies are needed to point out the specific genetic defect or alteration in purine metabolism pathway in these patients.

The present project highlights the role rs594445 in two psychiatric conditions and implies the presence of common genetic factors for these disorders. The underlying mechanism might be related to involvement of MOCOS in neurotransmission in developing and adult brain or its participation in regulation of purine metabolism. Our study has some limitations. First, we did not perform functional assays to appraise the effect of the nucleotide substitution on enzyme activity. Moreover, the sample size in BP2 and MDD subgroups were small. Therefore, the observed lack of association between rs594445 and these conditions may be due to small sample size.

## Data Availability Statement

The raw data supporting the conclusions of this article will be made available by the authors, without undue reservation, to any qualified researcher.

## Ethics Statement

The study protocol was approved by the Ethics Committees of Shahid Beheshti Universities of Medical Sciences. The patients/participants provided their written informed consent to participate in this study.

## Author Contributions

AS and FN performed the experiment. AK and MD analyzed the data. MT and SG-F wrote the draft and revised it. All authors contributed to the article and approved the submitted version.

## Conflict of Interest

The authors declare that the research was conducted in the absence of any commercial or financial relationships that could be construed as a potential conflict of interest. The reviewer, AS, declared a shared affiliation, with several of the authors, to the handling editor at the time of review.
